# Bio‐Inspired Neuromorphic Sensory Systems from Intelligent Perception to Nervetronics

**DOI:** 10.1002/advs.202409568

**Published:** 2024-11-11

**Authors:** Elvis K. Boahen, Hyukmin Kweon, Hayoung Oh, Ji Hong Kim, Hayoung Lim, Do Hwan Kim

**Affiliations:** ^1^ Department of Chemical Engineering Hanyang University Seoul 04763 Republic of Korea; ^2^ Institute of Nano Science and Technology Hanyang University Seoul 04763 Republic of Korea; ^3^ Clean‐Energy Research Institute Hanyang University Seoul 04763 Republic of Korea; ^4^ Present address: Department of Chemical Engineering Stanford University Stanford CA 94305 USA

**Keywords:** artificial synapse, bio‐inspired, machine learning, neuromorphic sensory system

## Abstract

Inspired by the extensive signal processing capabilities of the human nervous system, neuromorphic artificial sensory systems have emerged as a pivotal technology in advancing brain‐like computing for applications in humanoid robotics, prosthetics, and wearable technologies. These systems mimic the functionalities of the central and peripheral nervous systems through the integration of sensory synaptic devices and neural network algorithms, enabling external stimuli to be converted into actionable electrical signals. This review delves into the intricate relationship between synaptic device technologies and neural network processing algorithms, highlighting their mutual influence on artificial intelligence capabilities. This study explores the latest advancements in artificial synaptic properties triggered by various stimuli, including optical, auditory, mechanical, and chemical inputs, and their subsequent processing through artificial neural networks for applications in image recognition and multimodal pattern recognition. The discussion extends to the emulation of biological perception via artificial synapses and concludes with future perspectives and challenges in neuromorphic system development, emphasizing the need for a deeper understanding of neural network processing to innovate and refine these complex systems.

## Graphical abstract

This review explores neuromorphic artificial sensory systems inspired by the signal processing mechanism of the human nervous system. It provides a comprehensive analysis of the latest advancements in artificial synaptic devices triggered by various stimuli, including optical, auditory, mechanical, and chemical inputs, and their subsequent processing through artificial neural networks for various applications.

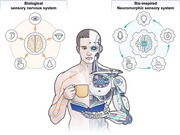

## 1 Introduction

**Figure 1:**
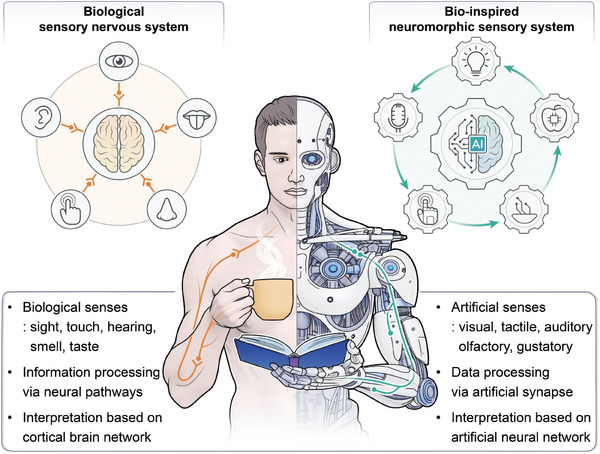
An illustration depicting a visual representation of both human and artificial signal processing pathways from various sensory inputs. The human nervous system consists of receptors, sensory nerves, and neurons for sensing, transmitting, and processing of sensory information (left part). Similarly, neuromorphic artificial sensory system mirroring the human nervous system, comprises sensing, transmission, and neural network algorithms for detecting and processing of sensory data (right part).

The human nervous system plays a crucial role in enabling signal transmission between different body parts and the brain, facilitating the reception and transmission of sensory data for coordinated actions.^[^ ^1^, ^2^, ^3^ ^]^ This system is categorized into the central nervous system (CNS, encompassing the brain and spinal cord) and the peripheral nervous system (PNS, including receptors, sensory, and motor nerves).^[^ ^4^, ^5^ ^]^ The PNS contains diverse sensory receptors (such as mechanoreceptors, photoreceptors, and nociceptors) that detect and react to environmental stimuli like light, sound, touch, and chemicals.^[^ ^6^ ^]^ These stimuli are then conveyed to the CNS through sensory nerves for neuronal computations, learning, cognition, and memory.^[^ ^7^ ^]^ The CNS interprets this sensory information and sends instructions to specific organs and tissues via motor nerve fibers to generate appropriate responses.^[^ ^6^ ^]^ This complex information processing involves an interconnected network of neurons and synapses, with presynaptic neurons generating nerve impulses (action potentials) in response to stimuli. These impulses are transmitted to postsynaptic neurons through synapses.^[^ ^8^ ^]^ The efficiency of impulse transmission between two neurons (known as synaptic weight) changes during neuronal activities. These changes in synaptic weight result in the modification of the strength of connections between the neurons (referred to as synaptic plasticity), facilitating short‐term plasticity (STP) and long‐term plasticity (LTP) depending on the retention time.^[^ ^4^, ^8^, ^9^ ^]^ STP enables synapses to execute essential computational tasks including signal encoding, filtering out extraneous signals, and facilitating decision‐making within the neural networks.^[^ ^10^, ^11^, ^12^ ^]^ Through adequate training, STP can transition into LTP by regulating the frequency and intensity of spikes (impulses) generated by presynaptic neurons.^[^ ^13^, ^14^, ^15^ ^]^ This process underpins the foundational mechanisms of learning and memory.^[^ ^5^, ^9^ ^]^ This complex system allows humans to perceive and interpret sensory inputs (**Figure** **1**, left side), through perceptual learning and adaptation.

Drawing inspiration from the sensory nervous system of humans (PNS and CNS), the development of neuromorphic artificial sensory (NAS) systems has garnered significant research interest due to their potential to create brain‐like computing systems that offer faster data processing and storage with low power consumption.^[^ ^8^, ^16^, ^17^ ^]^ Such advancement promises significant progress in next‐generation humanoid robotics, prosthetics, and wearable technologies.^[^ ^18^, ^19^ ^]^ NAS systems are primarily operated with three functionalities; sensory recognition (visual,^[^ ^20^, ^21^, ^22^ ^]^ tactile,^[^ ^23^, ^24^, ^25^ ^]^ auditory,^[^ ^26^, ^27^ ^]^ olfactory,^[^ ^28^, ^29^, ^30^ ^]^ and gustatory^[^ ^31^, ^32^ ^]^), neural signal processing,^[^ ^33^, ^34^, ^35^ ^]^ and interpretation of the sensory information based on neural network processing algorithms.^[^ ^21^, ^36^, ^37^ ^]^ NAS system transforms external sensory stimuli into electrical signals via artificial neurons and neural‐like devices, facilitating neural signal processing of external sensory information. Artificial neurons play a crucial role in bio‐inspired neuromorphic systems, emulating biological neural networks for bio‐sensing and bio‐interfacing applications. These neurons, particularly organic electrochemical neurons^[^ ^38^ ^]^ and organic artificial neurons (OANs),^[^ ^39^, ^40^ ^]^ mimic neuronal excitability and firing using organic mixed ionic‐electronic conductors. By interacting with biological carriers like ions and neurotransmitters, they closely replicate natural neuronal processes. OANs, for instance, exhibit spiking behavior modulated by the concentration of Na^+^ and K^+^ ions, allowing real‐time operation with biological tissues.^[^ ^39^, ^40^, ^41^ ^]^ In the context of bio‐sensing and bio‐interfacing, these neurons integrate sensors, oscillators, and synaptic transistors to convert physical stimuli into electrical impulses, mimicking action potentials in biological neurons. This capability is critical for applications like neuroprosthetics, biohybrid systems, and neuromorphic sensing. Their operation in biological environments makes them highly suitable for direct interaction with living tissues, paving the way for advancements in medical diagnostics, robotics, and neural interfaces, effectively linking artificial electronics with biological systems.^[^ ^39^ ^]^ It is noteworthy that the characteristics of input spike signals–their width, duration, amplitude, and frequency–vary depending on the specific type of sensory stimuli. Exploiting these sensory pulse signals, NAS system enables neural signal processing of sensory information, thereby facilitating the generation of synaptic memory signals, encompassing short‐term and long‐term memory (STM and LTM).

In recent research, NAS technologies have steadily progressed to augment artificial sensory neural network computation capabilities, providing brain‐like sensory information processing across various disciplines.^[^ ^42^ ^]^ Nevertheless, the predominant focus of the research on NAS systems has revolved around diverse materials for the synaptic device architecture, their sensing mechanisms across the five senses, and synaptic characteristics.^[^ ^4^, ^5^, ^43^, ^44^, ^45^ ^]^ Therefore, there exists an urgent necessity for a comprehensive exploration of neural network processing mechanisms for sensory information and its practical application, aiming to deepen our understanding of NAS systems and drive further advancements in the field. In this review, we highlight the representative NAS systems utilizing neural network algorithms to process synaptic sensory information in various applications. We begin with an analysis on the latest developments in artificial synaptic properties stimulated by optical, mechanical, sound, chemical, and hybrid inputs, focusing on elucidating their neural network processing capabilities for the sensory synaptic signals. Moreover, we delve into the cutting‐edge applications of NAS systems that replicate biological neuromorphic perceptions and neural interface behaviors, and finally, concluding with a discussion on the challenges and future directions for development of NAS technologies.

## 2 Artificial Sensory Neural Network Processing Algorithms

In order to realize brain‐like computation and interpretation, enabling tasks such as classification,^[^ ^46^ ^]^ image recognition,^[^ ^47^ ^]^ pattern recognition^[^ ^48^ ^]^ and prediction^[^ ^49^ ^]^ with remarkable precision, the integration of the sensory synaptic information with neural network algorithms stands as an essential prerequisite. Artificial Neural Network (ANN),^[^ ^50^, ^51^ ^]^ modeled after the biological neural network is composed of layers of nodes (neurons) connected by weights. Each neuron uses activation functions to process inputs and generate an output. ANN is adaptable in applications involving signal processing and sensor fusion due to its capacity to model non‐linear connections. Spiking Neural Network (SNN)^[^ ^51^, ^52^ ^]^ mimics the spiking behavior of neurons, where information is transmitted via discrete spikes over time. It is more ideal for neuromorphic computing platforms and offer efficient, event‐driven computation, making it well‐suited for energy‐efficient and real‐time sensory data processing. Both ANN and SNN are representative neural network algorithms, mimicking the structure of the brain and neural activity timing, respectively. Nonetheless, ANNs use continuous data processes, making them suitable for pattern recognition tasks requiring high accuracy. However, compared to ANNs, SNNs use event‐driven computation to process information via discrete spikes based on timing, making them efficient for real‐time tasks where response timing and energy efficiency are critical.^[^ ^50^, ^52^ ^]^ Deep Neural Networks (DNN)^[^ ^53^ ^]^ is a multi‐layered ANN structure that use deep layers of abstraction to extract complex features. In sensory systems, DNN employs unsupervised learning techniques, particularly effective for identifying and extracting features based on sensory data due to their ability to process high‐dimensional sensory inputs. Optic Neural Network (ONN)^[^ ^54^ ^]^ is a specialized neural network algorithm which takes a unique approach by utilizing optical signals for computations of high‐speed data processing and large‐scale pattern recognition from input data. This network integrates optical‐sensing capabilities with neural processing, offering high accuracy in pattern recognition tasks that involve optical inputs. Lastly, k‐Nearest Neighbors (KNN)^[^ ^55^ ^]^ is a non‐parametric algorithm that uses proximity to classify data by identifying similarities to the closet neighboring samples to a query point. It is particularly useful for classification and regression analysis. Each of these networks offers different strengths depending on the complexity and efficiency needs of the sensory application. Consequently, with these neural network algorithms, NAS system can facilitate the organization, identification, and interpretation of sensory information, empowering artificial intelligence (AI) and robotics with sophisticated recognition and decision‐making abilities with efficiency and accuracy (Figure 1, right side).

## 3 Neuromorphic Sensory System for Intelligent Perception

### 3.1 Artificial Visual Sensory System

**Figure 2:**
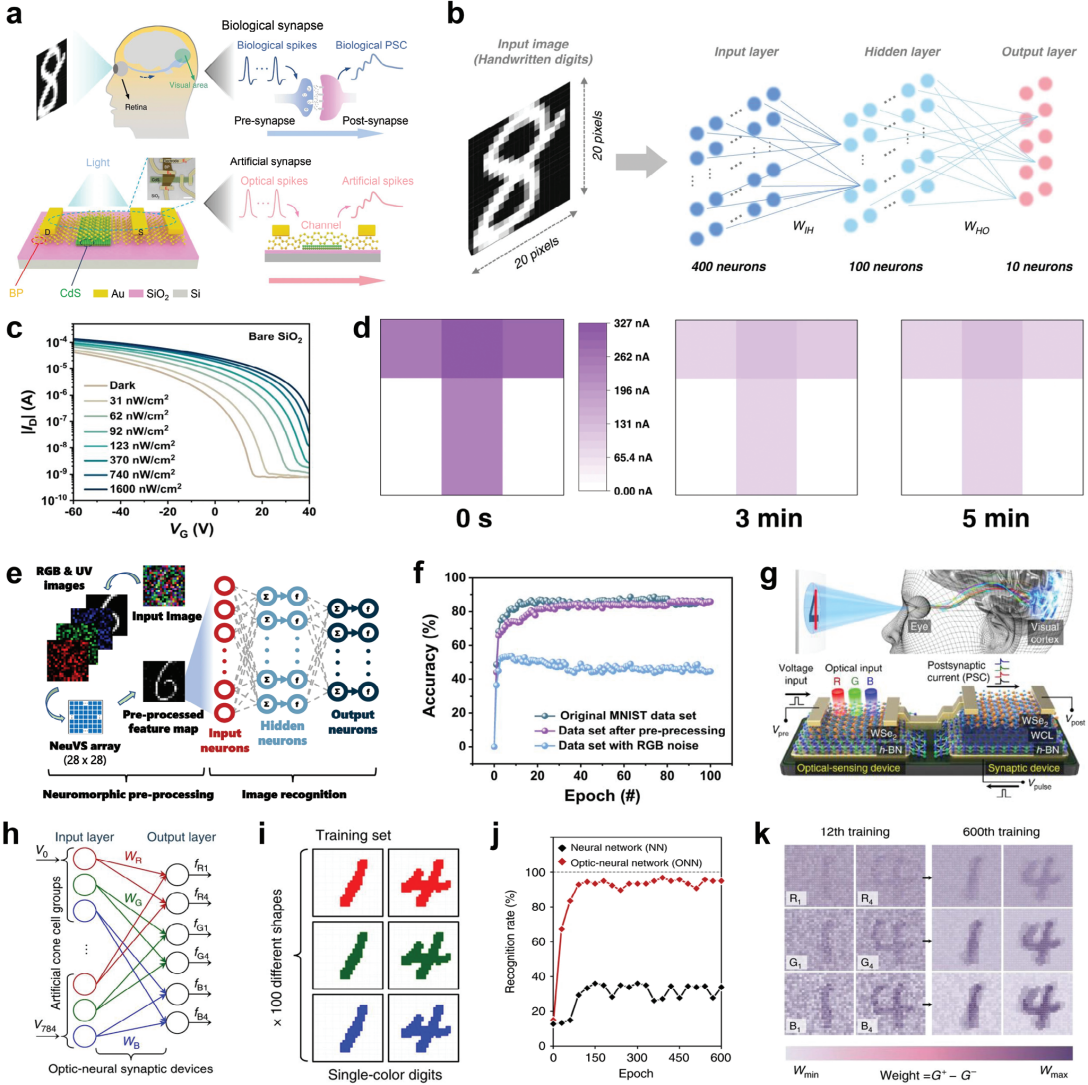
Artificial vision sensory systems based on synaptic devices a) Schematic illustration of visual signal transmission of the biological sensory system and artificial photonic synapse. b) Illustration of the simulated FONN for image recognition by the photonic synapse. c) Illumination intensities of the OPTs‐based tetrachromatic vision system. d) Detection and memorization of the letter T under UV light stimulation. e) Illustration of ANN structure for digit recognition. f) Comparing recognition accuracy with and without pre‐processing features. g) Schematic illustration of the h‐BN/WSe_2_ device integrated with a photodetector mirroring the human optic nerve system. h,i) Recognition of 28 × 28 RGB‐colored images based on the artificial ONN. j) Recognition rate as a function of training epochs with and without ONN. k) Weight mapping images after the 12th and 600th training epochs. (a,b) Reproduced with permission.^[^ ^58^ ^]^ Copyright 2022, Springer Nature. (c‐f) Reproduced with permission.^[^ ^47^ ^]^ Copyright 2023, Springer Nature. (g‐k) Reproduced with permission.^[^ ^54^ ^]^ Copyright 2018, Springer Nature.

Vision is paramount in the receiving and processing of information, undertaking the majority of external environmental information obtained by humans.^[^ ^56^ ^]^ The retina perceives and processes optical information, which is then sent to the visual cortex of the brain for processing and memory (**Figure** **2a**).^[^ ^57^, ^58^ ^]^ The development of artificial vision systems emulating the human visual sensory process constitutes a crucial advancement in the fields of humanoid robotics and computer vision. Such bioinspired artificial visual sensory systems can be realized by integrating phototransistors made from photoresponsive semiconductors to convert light into electrical signals. The phototransistors absorb incident light and convert it into electrical charges by generating electron‐hole pairs via charge trapping effect when exposed to light, triggering a flow of current proportional to the light intensity.^[^ ^4^, ^56^, ^59^ ^]^ An optoelectronic synapse that reacts to light stimulation serves as the fundamental component in replicating the visual sensory functions. As shown in Figure 2a, a black phosphorus (BP) and cadmium sulfide (CdS) heterostructure‐based artificial photonic synapse with remarkable light response and ultralow power consumption (4.78 fJ) has been demonstrated.^[^ ^58^ ^]^ The device responded to different light intensities and wavelengths through hole traps in CdS upon excitation of electrons owing to interface barrier between the BP and the CdS. Different wavelengths of light generated varying energy levels, causing electrons to jump across the band gap differently. Notably, a Fully‐Connected Optoelectronic Neural Network (FONN), a special type of ONN algorithm, was incorporated with the photonic synapse to recognize handwritten digits. The FONN received and processed optical signals corresponding to an input image (handwritten digit) through layers of artificial synapses which adjusted their conductance based on the intensity and wavelength of the incident light (Figure 2b). This adjustment simulated synaptic weight in the neural networks, thus enabling the network to effectively recognize patterns in the data, which results in accurate digit recognition.

Besides the performance of neuromorphic vision sensors at ultralow power consumption, it is vital for these sensors to perceive invisible UV light in addition to visible spectrum. This facilitates the exploitation of advanced artificial visual perception systems for improved medical diagnostics, complex image processing, and forensic applications. Inspired by the tetrachromatic vision system in some animals, a neuromorphic vision sensor based on organic phototransistors (OPTs) that can detect ultraweak UV light has been constructed.^[^ ^47^ ^]^ The sensor can detect UV light at wavelengths ≈370 nm and illumination intensities as low as 31 nW cm^−2^ (Figure 2c). Additionally, the OPTs exhibited photoresponsive conductivity changes, which enabled the detection and memorization of optical signals owing to high density charge‐trapping states. Using an OPT synaptic 3 × 3‐pixel array, the device detected and memorized the letter “T” when exposed to the image “T” under UV illumination (Figure 2d). This exposure caused a change in the conductance of the OPTs in the sensor array, effectively memorizing the image. Moreover, as shown in Figure 2e, ANN with a three‐layer structure (400 input neurons, 100 hidden neurons and 10 output neurons) was employed to analyze pre‐processed patterns obtained from the OPT array. The obtained output was a weighted average operation performed by the ANN convolutional kernel on the RGB and UV values of a single pixel, allowing it to distinguish and extract specific UV‐related information. The OPT array effectively extracted UV information, resulting in a high recognition accuracy like the original image dataset used to train the ANN (Figure 2f).

Beyond image and shape recognition, colored and color‐mixed patterns recognition play an important role in item identification, scene interpretation, and decision‐making.^[^ ^58^ ^]^ It is therefore crucial for neuromorphic vision systems to accurately recognize color and color‐mixed patterns, allowing them to perform more detailed and sophisticated tasks, such as differentiating between objects based on subtle color variations and providing more detailed data for various applications such as robotics, autonomous vehicles, and advanced surveillance systems. As shown in Figure 2g, an optic‐neural synaptic (ONS) device based on hexagonal boron nitride (h‐BN) and tungsten diselenide (WSe_2_) heterostructure was developed by integrating synaptic and optical‐sensing functions on the same device.^[^ ^54^ ^]^ The device exhibited different responses to the wavelengths of red (R), green (G), and blue (B) light owing to trapping and de‐trapping of electrons in a weight control layer (WCL) generated by O_2_ plasma‐treatment on h‐BN. The ONS device was integrated into an ONN for colored and color‐mixed pattern recognition. As shown in Figure 2h, the ONN comprised of three neurons and group cells consisting of a 28 × 28 array, where each group cell was connected to RGB‐colored and color‐mixed patterns 1 and 4, resulting in six output classifications (Figure 2i). The ONN effectively identified both colored and color‐mixed numbers with a high recognition accuracy rate (>90%) compared to conventional neural network (without optical‐sensing function), that exhibited a low accuracy rate (<40%) (Figure 2j). With the increase of training epochs, the synaptic weights associated with the patterns grew richer notable after the 12th and 600th training epochs (conspicuous in blue patterns because the device responds stronger to blue light), as depicted in Figure 2k. This enhancement was attributed to the effective optimization of RGB‐color‐mixed numerical pattern recognition by the ONS device. By closely emulating human visual and cognitive processes, these technologies provide the foundation for future work toward building intelligent sensors, robotics, and advanced neuromorphic computing systems that integrate optical‐sensing and synaptic functionalities for highly integrated complex pattern recognition tasks.

### 3.2 Artificial Tactile Sensory System

**Figure 3:**
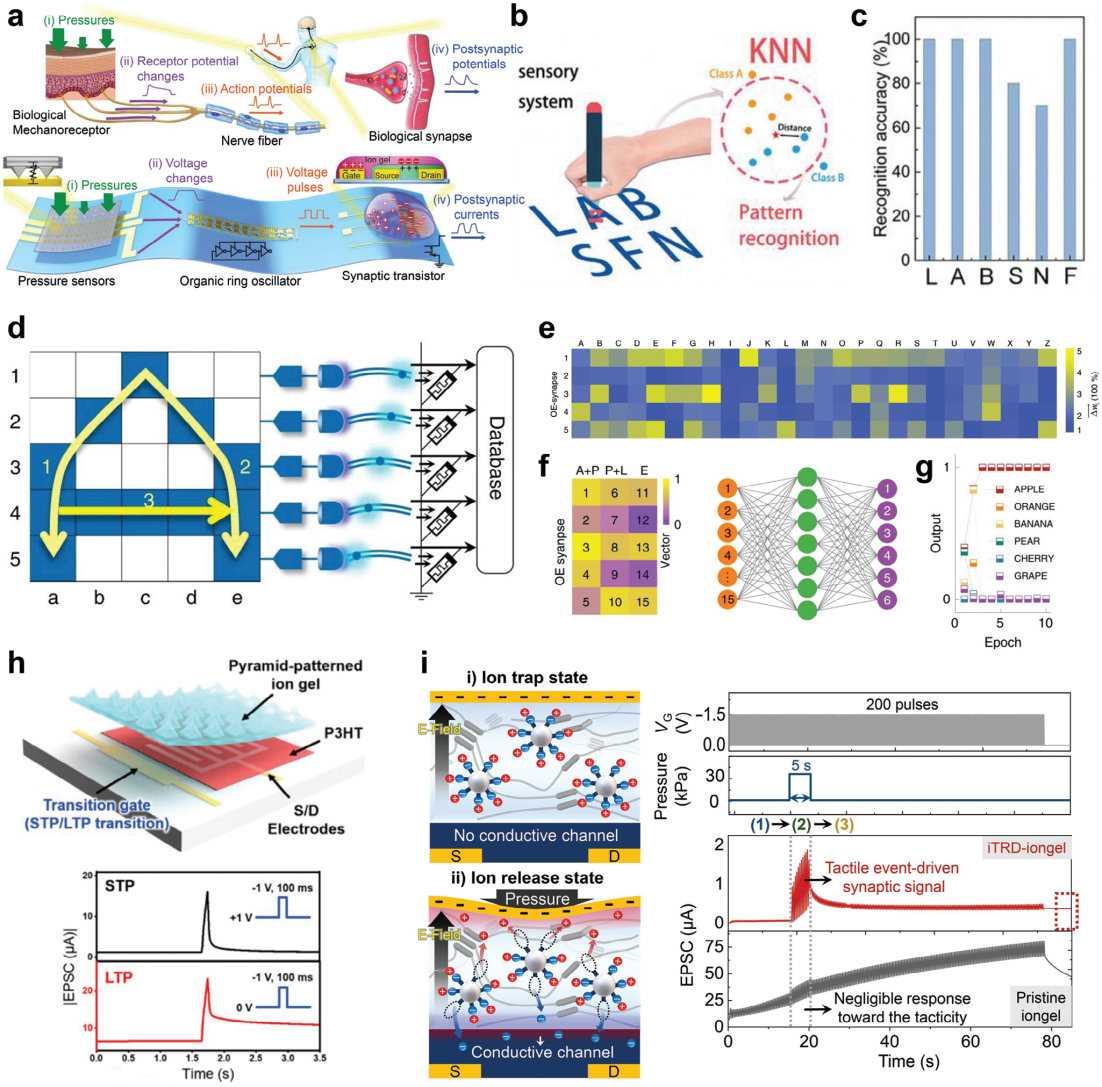
Artificial tactile sensory systems based on synaptic devices a) Configuration of artificial sensory nerve comprising of pressure sensors, ring oscillator, and synaptic transistor in comparison with a biological sensory system. b) Illustration of the writing of English letters by the sensory system on a “pen”, and the processing of collected data by the KNN algorithm to identify different letters. c) Recognition accuracy of each English letter. d) Illustration of the structure and working mechanism of the optoelectronic SNN. e) Weight changes depicting the learning and memory capabilities for the handwritten letters of the alphabet. f,g) Dimensionality reduction of the vector representing “APPLE” using artificial neural network and the training results depicting the recognition of various words. h) OECT‐based artificial tactile system that integrates tactile perception, both STP and STP, and learning functionalities within a single device. i) Ion trap and release in NeuroMAT and tactile perception performance under applied tactile stimulation. (a) Reproduced with permission.^[^ ^41^ ^]^ Copyright 2018, American Association for the Advancement of Science. (b,c) Reproduced with permission.^[^ ^48^ ^]^ Copyright 2019, Wiley‐VCH. (d‐g) Reproduced with permission.^[^ ^46^ ^]^ Copyright 2020, Springer Nature. (h) Reproduced with permission from.^[^ ^66^ ^]^ Copyright 2020 American Chemical Society. (i) Reproduced with permission.^[^ ^68^ ^]^ Copyright 2023, American Association for the Advancement of Science.

Humans perceive tactile stimuli through the interplay of pressure signals and vibrations, caused by spatiotemporal mechanical changes on the skin, facilitating the recognition of shape, texture, weight, and other environmental physical characteristics.^[^ ^60^, ^61^ ^]^ In biological sensory mechanisms, mechanoreceptors detect external tactile information that are transformed into action potentials via somatosensory transduction and the resulting impulses are transmitted to motor (efferent) nerves, forming a biological reflex arc (**Figure** **3a**).^[^ ^41^, ^62^ ^]^ The development of artificial tactile perception systems that mimic the sensory and signal processing functions of the biological tactile sensory nervous system holds great potential for use in robotics, medical devices, prosthetics, and electronic skin (e‐skin).^[^ ^63^, ^64^, ^65^ ^]^ Such bio‐inspired tactile sensory systems can be realized by integrating pressure sensors with iontronic synaptic transistors. External tactile information from the pressure sensor is converted into voltage pulses via artificial nerve fiber (ring oscillator or cable). The ring oscillator provides a viable option as artificial nerve fiber due to its robustness to voltage degradation and parasitic resistances, ensuring reliable conversion and transmission of tactile information. Likewise, the cable serves as a direct and reliable pathway for sensory information from the pressure sensor to the synaptic transistor. These electrical signals from the artificial nerve fibers are subsequently integrated and transformed into PSCs by the synaptic transistor (Figure 3a).^[^ ^41^ ^]^ To achieve highly sensitive pressure sensor, surface modification (such as pyramid structure) of active matrixes or electrodes has been utilized.^[^ ^41^, ^48^, ^66^, ^67^ ^]^ The pyramid structure minimized the initial contact area between the active matrix and electrodes, reducing the conductivity of the device. Under external pressure, the spires of the pyramids contacted the electrodes increasing the contact areas, leading to an increase in output current. The difference in the initial and final resistance or capacitance resulted in higher sensitivity of the pressure sensor device. Using a pyramidal piezoresistive sensor and a nafion‐based memristor, a biomimetic tactile sensory nerve system has been developed (Figure 3b).^[^ ^48^ ^]^ This system processed external mechanical stimuli information emanating from the sensor via the memristor, acting as artificial synapse. The memristor processed and memorized tactile information using electric‐field‐driven drift and concentration‐gradient‐driven diffusion of protons. Under positive bias, protons migrated along the electric field, enhancing the conductive channel within the nafion film. This enabled the memristor to stimulate synaptic functions by modulating the conductance state in response to stimuli. Thus, the integrated system can accurately detect, process, and memorize tactile information, characterized by varying levels of intensity, frequency, duration, and speed. As a proof of concept, a smart “pen” equipped with the artificial sensory nerve system in conjunction with KNN algorithm, was used to recognize handwritten English letters. As shown in Figure 3b, the smart “pen” recognized letters by producing electrical signals under mechanical pressure, that varied in peak shapes depending on the letter being written, owing to the distinct strokes and pulse duration required for each letter. After 10‐time training of the KNN, the system achieved an average accuracy of 91.7% in recognizing six different characters (Figure 3c).

The connection of artificial nerve fibers with synaptic devices via physical contact typically leads to not only reduced electrical signal transmission efficiency due to the inherent contact resistance between the components but also complicated fabrication. Moreover, the precise understanding of complex tactile information encompassing variations in pressure at distinct times necessitates the dual encoding of stimulus intensity and duration through rate coding, along with the precise timing of spikes via temporal coding. To address these challenges and enhance the recognition of mechanical stimuli, an innovative optoelectronic spiking afferent nerve system has been developed.^[^ ^46^ ^]^ This system featured a non‐contact integration of MXene‐based sensors with synaptic photomemristors and implemented both rate and temporal coding. Such integration enabled a more detailed representation and understanding of complex tactile information. The pressure information was converted into optical spikes via an analog‐to‐digital conversion (ADC) mechanism coupled by light emitting diode (LED) circuits, enabling non‐contact signal transmission. The spikes were then processed by the synaptic photomemristor, enabling the integration of light pulses into PSCs and implementation of both rate and temporal coding. Through dimensionality‐reduced feature extraction and learning, this system demonstrated remarkable capabilities in recognizing and memorizing handwritten alphabets and words. As shown in Figure 3d, a 5 × 5 sensor array connected to ADC‐LED and synaptic photomemristors was utilized to recognize handwritten letters by training. The architecture simplified the recognition process by reducing the dimensionality of data from 25 to 5 dimensions, based on the spiking proportions of the photomemristors. This reduction transformed each letter into a distinct 5D vector, forming a dictionary for the alphabet (Figure 3e). Additionally, an artificial neural network with inputs and outputs corresponding to dimensionality‐reduced vectors (by combining vectors of two subsequent letters), was trained to classify handwritten words such as “APPLE”, “ORANGE”, “BANANA” and others (Figure 3f,g).

Despite the high accuracy of tactile information recognition, the integration of separate sensors and synaptic devices leads to a complex design architecture, energy burdens, and data latency. In addition, the characteristics of LTM are relatively inferior because the voltage spike is a tactile event‐driven, thereby restricting the provision of sufficient voltage potential for deep ion penetration into semiconductor layers. To overcome this, efforts to develop monolithic artificial tactile nerve, which do not physically separate components (i.e., tactile sensor, artificial nerve fiber, and synaptic devices), while possessing robust LTM functionalities, are in progress. Recently, a dual‐gated organic electrochemical transistor (OECT) with a pyramid‐ patterned iongel, which integrates tactile perception, memory, and learning functionalities within a single device, has been developed (Figure 3h).^[^ ^66^ ^]^ When external pressure and negative voltage were applied on the iongel, the conductance of the OECT can be modified via electrochemical doping triggered by anion penetration into a p‐type polymer semiconductor layer. The additional applied voltage from a transition gate electrode can modulate the duration time of the conductance by controlling the diffuse out characteristics of the penetrated anions, resulting in a facile transition between STP and LTP functionalities. However, this device still exhibited weak tactile memory retention properties (typically < 200 s) due to an intrinsically electrochemical equilibrium behavior of the iongel. To address the weak tactile memory, an ion trap and release dynamics‐driven iongel (iTRD‐iongel) has been designed to facilitate high tactile sensitivity and enhanced tactile memory retention in a monolithic artificial tactile neuron (Figure 3i).^[^ ^68^ ^]^ In the iTRD‐iongel, ions are initially trapped to silica microparticles within the iongel, in which the electrochemical properties are not modulated by an external electrical field. Depending on pressure information (e.g., magnitude, frequency, and duration), some of the trapped ions are released, converting into free ions that can be modulated by the electric field. Consequently, these released ions can deeply penetrate into the semiconductor layer through additional voltage pulses, which are independent of tactile events, thereby generating highly robust tactile memory (> 3680 s).

The artificial tactile sensory systems discussed in this section have successfully realized human‐like tactile perception and computation. These capabilities could be utilized in prosthetics, intelligent robotics, and neuromorphic e‐skin with enhanced recognition and interpretation of surrounding environments. Further advanced applications of these technologies will be discussed in a later section.

### 3.3 Artificial Auditory Sensory System

**Figure 4:**
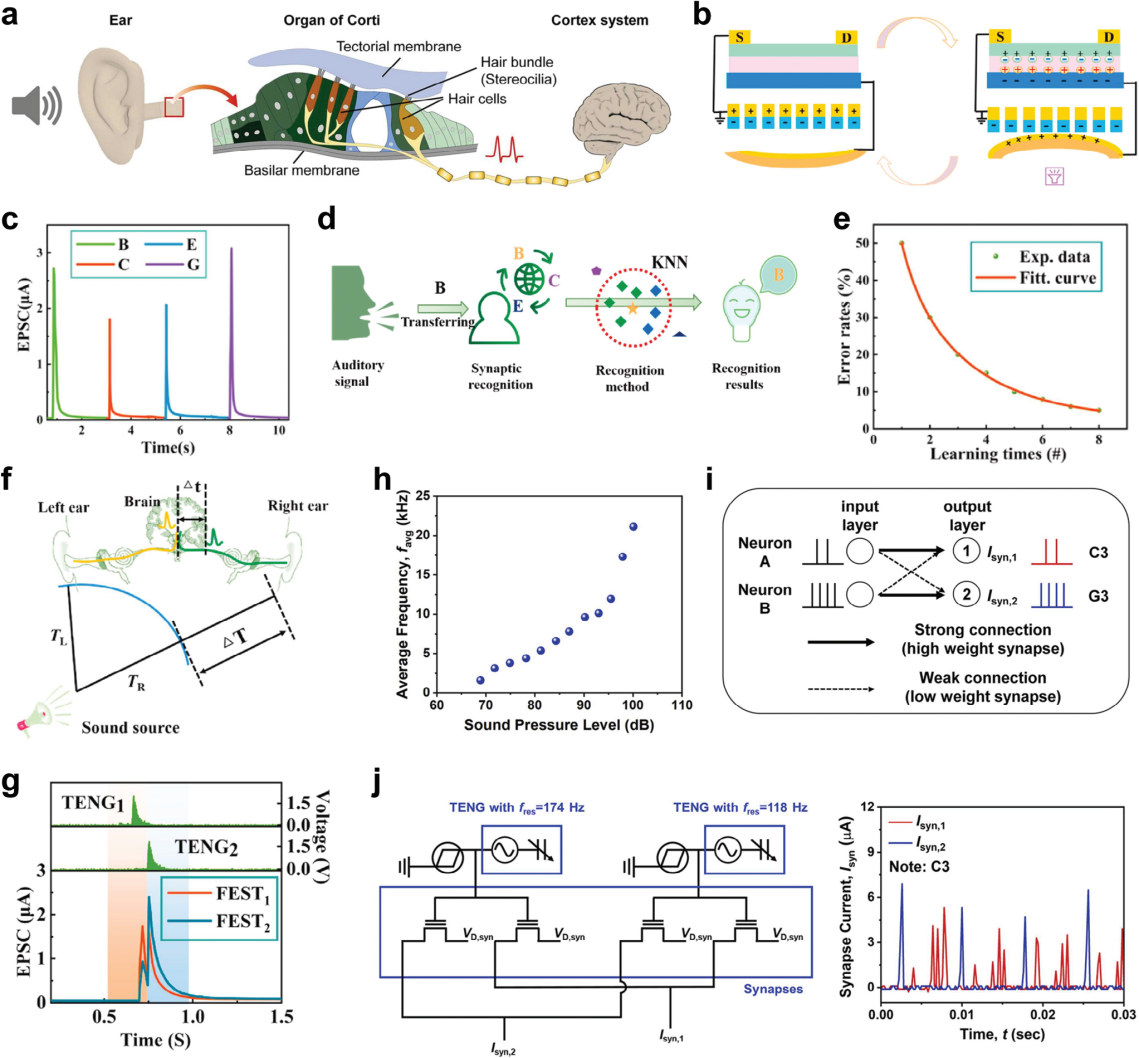
Artificial auditory sensory systems based on synaptic devices a) Auditory signal transmission of the biological sensory system. b) Operation mechanism of TENG actuated artificial auditory system. c) Distinguishing different words based on EPSC amplitude. d,e) Acoustic signal recognition by KNN algorithm. f,g) Detection and recognition of sound location using an ANN algorithm. h) Spiking frequency dependency on sound pressure level. i,j) Musical pitch classification through SNN algorithm with single‐layer perceptron. (a) Reproduced with permission.^[^ ^70^ ^]^ Copyright 2023, Elsevier Ltd. (b‐g) Reproduced with permission.^[^ ^26^ ^]^ Copyright 2020, Elsevier Ltd. (h‐j) Reproduced with permission.^[^ ^70^ ^]^ Copyright 2023, Elsevier Ltd.

In conjunction with the visual and tactile system, the auditory modality is one of the crucial sensory capabilities for understanding and interacting with the external world. The human auditory system has a complex signal transduction pathway capable of detecting sound wave frequencies ranging from 20 to 20,000 Hz with superior sensitivity.^[^ ^69^ ^]^ Sound could generate mechanical signals in the form of vibrations, which transmit along the eardrum of the ears that play a key role in signal transmission and amplification, causing the fluid within the cochlea to vibrate (**Figure** **4a**).^[^ ^70^ ^]^ Acoustic signal is converted into electrical action potentials by stimulating hair cells due to vibrations of the fluid in the ear.^[^ ^71^ ^]^ The electrical signals are transmitted in the form of spikes to auditory cortex in the brain and they can be perceived as sounds. Therefore, based on the auditory sensory system, humans recognize sound through interaction between external stimuli and the brain to obtain significant functions such as a warning in dangerous situations and efficient communication with their surroundings. However, people who have impaired auditory sensory system experience communication difficulty due to information loss. To solve this problem, artificial acoustic sensors that emulate the biological auditory system have been consistently reported,^[^ ^72^, ^73^, ^74^, ^75^ ^]^ and characteristics such as sound detection accuracy, sensing range–their frequencies and amplitudes, conformability with skin, and power consumption–are key requirements. Among reported devices, piezoelectric materials have been widely used in acoustic sensors^[^ ^36^, ^76^, ^77^, ^78^ ^]^ because they exhibit low energy consumption due to their ability to directly convert mechanical signals into electrical signals similar to biological system.^[^ ^77^, ^78^, ^79^ ^]^ While high performance in terms of sound energy harvesting can be exhibited, low electrical output is often obtained due to the limitations in signal conversion efficiency.

As an alternative, the triboelectric nanogenerator (TENG), which can achieve a high electrical output while being self‐powered through triboelectrification and electrostatic induction effect, has emerged as a promising candidate.^[^ ^80^, ^81^, ^82^, ^83^ ^]^ For instance, a proposed ultrathin acoustic sensor based on flexible nanofibers featuring a wide frequency response (20–5000 Hz) and an ultra‐sensitive response (110 mV/dB) has been constructed using a triboelectric cochlea device that incorporates an annular inner boundary architecture.^[^ ^84^ ^]^ The operational mechanism of the TENG as an acoustic receptor, which enables self‐powering, involves the following steps^[^ ^26^ ^]^: i) one of the materials, possessing different triboelectric properties, vibrates under acoustic pressure, leading to triboelectrification when materials come into contact ii) upon separation, charge redistribution occurs as the materials move apart, and free electrons flow between the electrodes until electrostatic equilibrium was reached. As a result, the movement of charges induced by the acoustic waves between the two electrodes led to the generation and output of an electrical signal (Figure 4b). These electrical signals were then transmitted to an additional circuit or amplifier, where they were utilized to analyze sound characteristics such as decibel level, frequency, and pattern.

Moreover, an artificial auditory system that captures, processes, and memorizes large‐scale acoustic information was developed. This artificial auditory system's architecture consisted of a self‐adaptive and self‐powered artificial auditory pathway, comprising TENG and field effect synaptic transistor (FEST) as acoustic receptors and synapses, respectively.^[^ ^26^ ^]^ The TENG converted external voice signals into electrical signals which were transmitted to the FEST for processing and generation of excitatory postsynaptic current (EPSC), mimicking synaptic behavior in biological systems. The EPSC amplitude changed in response to different sound frequencies and intensities, which were crucial for recognizing distinct acoustic signals correlated with the word commands (B, C, E, G) received from the acoustic receptors (Figure 4c). The KNN algorithm, which classifies new data according to the trends of adjacent data, was employed to classify and identify word instructions accurately based on the EPSC data (Figure 4d). By training the model with a set of acoustic data, the KNN algorithm refines its classification accuracy, achieving up to 95% recognition accuracy after eight training cycles (Figure 4e).

In addition to recognizing specific auditory signals, identifying the location of sound is crucial for enhancing the ability to recognize situations and events in the surrounding environment.^[^ ^85^ ^]^ For example, assistive devices for hearing impaired individuals enable them to perceive the location of warning sounds, helping them avoid potentially dangerous situations. The human brain determines sound location by detecting differences in synaptic currents (Δt), which depends on the interaural time difference (ΔT) (Figure 4f).^[^ ^26^, ^86^ ^]^ In the proposed bio‐inspired system, the direction of the sound source was ascertained using a neural network consisting of multiple FESTs. When sound is received by the system, the difference in EPSC amplitudes between the two artificial auditory pathways that corresponded to the left and right ears helped identify the direction of the sound source, effectively enabling spatial hearing (Figure 4g).^[^ ^26^ ^]^ Furthermore, a self‐aware artificial auditory neuron module, integrating TENG as an auditory sensor and a bi‐stable resistor (biristor) as artificial neuron, has been constructed.^[^ ^70^ ^]^ Incident sound waves that reached the TENG exerted a corresponding sound pressure, generating alternating current (AC) which was transmitted to the biristor neuron (Figure 4h). The biristor operates on the principle of leaky integrate‐and‐fire, converting the AC input into spiking voltage output that that was processed through SNN for sound classification. For musical pitch classification, the system utilized a single‐layer perceptron (SLP) neural network with two artificial auditory neuron modules, each sensitive to different frequencies, corresponding to distinct piano pitches, C3 and G3 (Figure 4i). Following a winner‐takes‐all rule, the pitch classification was determined by the connection strength (or synaptic weight) of the spiking signals of the neuron module that was transferred to the transistor‐type artificial synapses (Figure 4j). The SLP distinguished between the two distinct piano notes by comparing the synaptic output currents. Consequently, C3, which exhibited a higher frequency output current, can be detected. This capability is significant because it allows the SNN‐based artificial auditory system to accurately identify sounds that are challenging for humans to differentiate. These systems show promise for applications in neuromorphic auditory processing and human‐computer interaction in noisy environments.

### 3.4 Artificial Olfactory and Gustatory Sensory System

**Figure 5:**
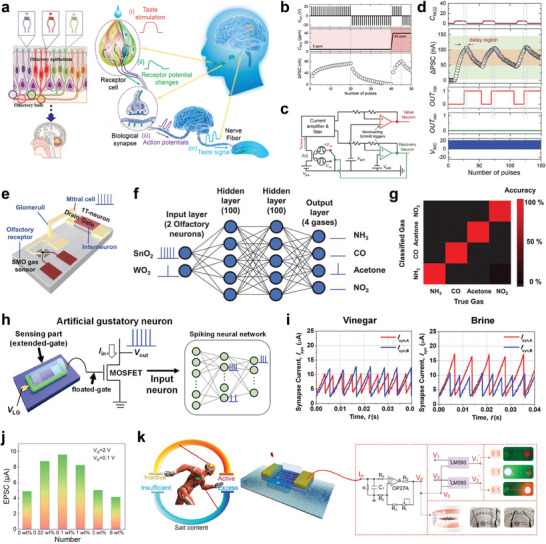
Artificial chemical sensory systems based on synaptic devices a) Schematics of biological olfactory and gustatory system and signal transmission pathways. b) LTP/LTD characteristics by NO_2_ concentrations (5 and 40 ppm). c,d) Circuit diagram of feedback‐controlled response system and operation characteristics under NO_2_ environment. e) Schematic illustration of artificial neuron module device consisting of SMO gas sensor and a single transistor neuron. f,g) Classification of four gas types (acetones, ammonia, carbon monoxide, NO_2_) by SNN algorithm. h) Structure of MOSFET‐based artificial gustatory neuron. i) Identification of two liquids (Vinegar and Brine) by comparing spiking frequency of synaptic currents. j) EPSC under different concentrations of salt solution. k) Excessive‐intake warning system utilizing light indicator activated by salt concentration conditions. (a) Reproduced with permission.^[^ ^89^ ^]^ Copyright 2016, Springer Nature; Reproduced with permission.^[^ ^107^ ^]^ Copyright 2022, American Chemical Society. (b‐d) Reproduced with permission.^[^ ^105^ ^]^ Copyright 2022, Wiley‐VCH. (e‐g) Reproduced with permission.^[^ ^28^ ^]^ Copyright 2022, Wiley‐VCH. (h,i) Reproduced with permission.^[^ ^114^ ^]^ Copyright 2022, American Chemical Society. (j,k) Reproduced with permission.^[^ ^107^ ^]^ Copyright 2022, American Chemical Society.

Unlike other sensory modalities, biological olfactory and gustatory sensory systems are equipped with chemical receptors that serve as principal conduits for conveying taste and odor information to the brain.^[^ ^87^, ^88^ ^]^ Although these two systems possess distinct structures, they share a common functional mechanism wherein chemical molecules–odorants and tastants–enter the sensory organ and bind to receptors. The olfactory system consists of the olfactory receptors, olfactory bulb, and olfactory nerves, where odorants reach olfactory epithelium in the nasal orifice and interact with various types of olfactory receptors (**Figure** **5a**).^[^ ^89^ ^]^ Approximately 400 distinct olfactory receptors play a pivotal role in olfaction, enabling humans to detect over 10,000 odors through the interaction between odorants and these receptors.^[^ ^89^, ^90^, ^91^ ^]^ The human olfactory system offers a model for the development of artificial olfactory systems based on field‐effect transistors (FET),^[^ ^92^, ^93^, ^94^ ^]^ memristors^[^ ^95^, ^96^ ^]^ and electrochemical devices.^[^ ^97^, ^98^, ^99^ ^]^ These systems known as electronic noses (e‐noses) are capable of recognizing chemical stimuli such as hydrogen sulfide,^[^ ^95^, ^100^ ^]^ nitrogen dioxide (NO_2_)^[^ ^101^, ^102^ ^]^ and volatile organic compounds,^[^ ^103^, ^104^ ^]^ enabling the monitoring of air quality levels and detection of respiratory disorders such as lung cancer.

Recently, a feedback‐controlled response system based on artificial sensory synapse for detecting high levels of NO_2_ gas has been developed.^[^ ^105^ ^]^ The artificial sensory synapse was built from an organic heterostructure consisting of a charge trapping layer (pentacene) and a hole‐conducting layer (copper‐phthalocyanine). When exposed to NO_2_ gas, the NO_2_ molecules interact with the pentacene layer, inducing electrons trapping effect and subsequent accumulation of charges in the hole‐conducting layer. This occurred due to the electron‐withdrawing nature of NO_2_, adjusting the conductance of the synaptic device, allowing the device to exhibit EPSC and inhibitory postsynaptic current depending on the concentration of NO_2_ (Figure 5b). The sensory synapse was connected to two artificial neuron circuits (valve and reconvery neurons) in the feedback‐controlled response system to process and respond to varying NO_2_ concentrations (Figure 5c). Upon detecting NO_2_ gas, the sensory synapse adjusted its conductance based on the concentration of the gas. Changes in the PSC above the upper threshold of 100 nA activated the first neuron circuit, which controlled an air valve to purge the chamber till the concentration of NO_2_ reduced below the risk level indicated by the lower threshold of 50 nA (Figure 5d). On the other hand, a swift change in the PSC above 150 nA engaged the second neuron circuit which triggerred an alarm and initiated a negative voltage pulses to reset the sensor synapse, effectively responding to elevated levels of NO_2_ exposure. The capability of the system to detect varying concentration of NO_2_ and initiate the appropriate responses demonstrates its successful simulation of the efficient risk‐response system of the human nervous system.

Similarly, an innovative artificial olfactory neuron module for e‐nose which integrates a chemoresistive gas sensor has been reported.^[^ ^28^ ^]^ The device based on a metal‐oxide‐semiconductor FET (MOSFET) was constructed from a semiconductor metal oxide (SMO) and a single transistor neuron (1T‐neuron) (Figure 5e). The SMO sensor was composed of tin(IV) oxide (SnO_2_) and tungsten(VI) oxide (WO_3_) as sensing materials, which detected various gases (acetones, ammonia, carbon monoxide, and NO_2_) due to changes in its resistance. The integration of the SMO sensor and 1T‐neuron enabled in‐sensor classification of different gases using SNN algorithm. As presented in Figure 5f, the SNN, composed of 2 input neurons, 100 hidden neurons in two hidden layers each, and 4 output neurons, classified different gases by processing the input spikes generated by the artificial olfactory neuron. The 2 input neurons, corresponding to the SnO_2_ and WO_3_ gas sensors, transmitted the spiking frequencies modulated by the gas concentration to the 1T‐neuron. When the output spikes from the 1T‐neuron reached the firing threshold of 5.3 V, they were propagated through the two hidden layers through synapse crossbars. This process was repeated until the output neurons (each corresponding to a particular gas), spiked to signal the identification of the classified gas species with an accuracy of 98.25% (Figure 5g). The high accuracy of neuromorphic e‐noses demonstrated their potential applications in monitoring air quality, detecting various toxic gases, and early diagnosis of respiratory diseases.

The biological gustatory sensory system not only plays a crucial role in determining human nutritional status but also influences emotional responses such as the pleasure and displeasure associated with food consumption.^[^ ^106^ ^]^ This system detects food tastes via the taste receptor cells located in taste buds at the front of the tongue (Figure 5a).^[^ ^107^ ^]^ When tastants interact with the taste buds, the cells activate gustatory receptors, each attuned to one of the five primary tastes: sweet, bitter, sour, salty, and umami.^[^ ^108^, ^109^ ^]^ This interaction triggers the release of neurotransmitters due to changes in membrane (receptor) potential or intracellular calcium ion concentrations.^[^ ^106^ ^]^ The neurotransmitters, carrying taste information, are transmitted through numerous synapses to the gustatory cortex of the brain, enabling humans to recognize tastes patterns.^[^ ^106^, ^107^ ^]^ Mimicking the human gustatory sensory systems, several aritificial tongues capable of detecting and discriminating between various tastants have been reported.^[^ ^110^, ^111^, ^112^, ^113^, ^114^ ^]^ Notably, an innovative MOSFET‐based artificial gustatory neuron has been developed into an electronic tongue (e‐tongue) to identify the concentrations of chemicals (Figure 5h).^[^ ^114^ ^]^ The e‐tongue comprised pH‐ and sodium ion‐sensitive neurons which tranduced the ion concentrations into surface potential changes, thereby altering the threshold voltage and spiking frequency of the MOSFET. The system integrated an SLP to classify vinegar and brine using an SNN algorithm. The SNN distinguished between the vinegar and brine by analyzing the spiking frequency of synaptic currents, *I_syn,A_* and *I_syn,B_*. For vinegar, the pH‐sensitive neuron produced a higher spiking frequency in *I_syn,A_* whereas for brine, the sodium‐sensitive neuron produced a higher spiking frequency in *I_syn,B_* (Figure 5i). Thus, the classification of these liquids were based on the comparison of the spiking frequencies to determine the identity of the liquids.

Excessive consumption of food addictive such as salt and sugar can increase the risk of hypertension, cardiovascular diseases, and diabetes.^[^ ^107^, ^115^ ^]^ It is therefore, essential to modulate the intake of these food addictives to minimize the risks associated with excessive consumption. Developing a device capable of detecting and alerting excessive salt and sugar levels in food would contribute significantly to mitigating these health risks. In this regard, a neuromorphic gustatory system that monitors concentrations of sodium chloride (NaCl)‐a primarily composition of salt‐ and issue warnings has been built (Figure 5j,k).^[^ ^107^ ^]^ This system was composed of chitosan‐based iongel sensor, SnO_2_ nanowire synaptic device, and an effect‐executive unit. The flow of NaCl solutions through the iongel generated a difference in ion conductance due to varying concentration of Na^+^, causing corresponding changes in the EPSC values from the synaptic device (Figure 5j). The spike values served as control signals transmitted to the effect‐executive unit, which then provided appropriate feedback such as a warning about excessive salt intake. For a high NaCl concentrations (6 wt.%), the system issued a warning by activating a red light. Meanwhile, for appropriate NaCl concentrations (1 wt.%), the system turned on green light, indicating safer concentration levels (Figure 5k). This demonstration of artificial tongues proved the potential of combining neuromorphic engineering with biosensing to realize advanced sensory systems for various applications, including fresh food monitoring, sweat analysis, and medical diagnostics.

## 4 Multimodal and Cross‐Modal Artificial Sensory System

NAS system that relies on a singular sensory input for decision‐making often encounters unavoidable ambiguities owing to limited scope of the signal processing.^[^ ^6^ ^]^ To address these limitations, a plausible solution is to develop integrated multisensory computing fusion. Multisensory neural network systems that can integrate and process two or more stimuli simultaneously are fundamental to accurate perception and better understanding of the multimodal world. Compared to unimodal devices, these systems can synergize multiple perceptions such as vision, touch, smell, taste, and hearing, enabling AI and robots to perform complex recognition and decision‐making tasks in unfamiliar situations.^[^ ^49^ ^]^ This facilitates the capability of AI to emulate the learning and memory process of the human brain. Consequently, a number of bio‐inspired multimodal artificial sensory systems, emulating the multifunctional perception and processing of the human neural network, have been reported.

**Figure 6:**
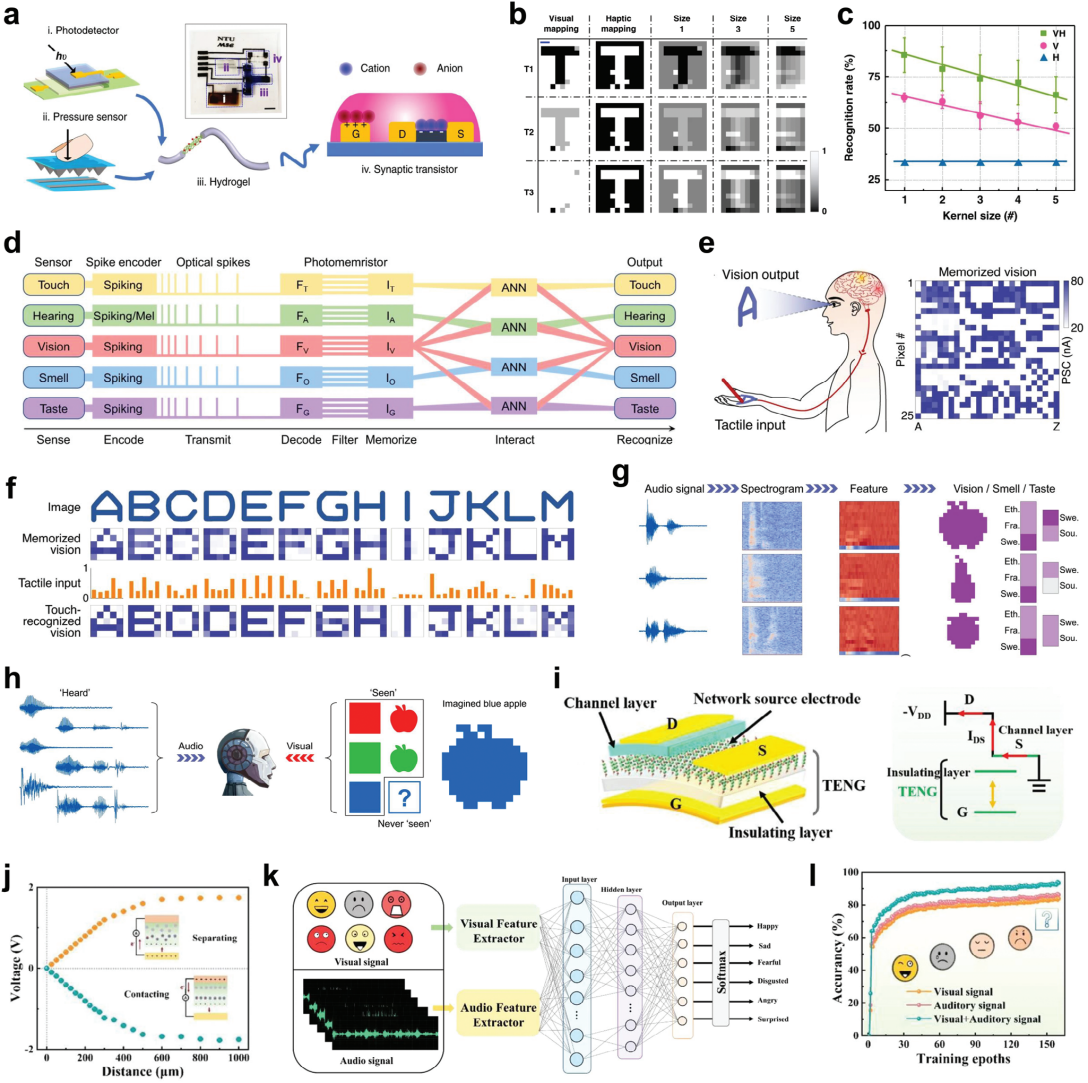
Multimodal NAS systems a) Configuration of bimodal sensory system with visual‐haptic (VH) fusion b) Multi‐transparency pattern recognition based on unimodal information (visual or haptic) and VH fusion information. c) Recognition accuracy of results based on unimodal and VH fusion information. d) Illustration of the structure and operation of the artificial MSeNN. e) Schematics of tactile input recognition, visualization, and memorization. f) Images, vision memory, and recognition of handwritten alphabet letters A‐M h) Recognized and reproduced images, smell and tastes of apple, pear, and blueberry upon associated audio input. i) Schematic illustration of the self‐powered VTT. j) Output voltage of the TENG as a function of distance. k) Recognition of six emotions (anger, fear, disgust, happiness, surprise, and sadness) by the multimodal emotion recognition system. l) Comparing emotion recognition accuracy at different input models. (a‐c) Reproduced with permission.^[^ ^116^ ^]^ Copyright 2020, Springer Nature. (d‐h) Reproduced with permission.^[^ ^49^ ^]^ Copyright 2021, Springer Nature. (i‐l) Reproduced with permission.^[^ ^118^ ^]^ Copyright 2022, Springer Nature.

As shown in **Figure** **6a**, to realize visual‐tactile integration, a bimodal artificial sensory neuron has been developed.^[^ ^116^ ^]^ This neuron incorporated a perovskite‐based photosensor and a resistive pressure sensor, which were connected through a hydrogel‐based ionic cable to an iontronic synaptic transistor. The signals from each sensor were transmitted to the transistor via the ionic cable and transduced into a transient channel current, like the biological EPSC. Notably, closely aligned visual‐tactile data enhanced the ability of the sensory fusion matrices to recognize alphabetic patterns with varying levels of transparency. To achieve recognition of the multi‐transparent patterns, both unimodal and bimodal matrices with a 10 × 10 pixels arrangement were designed. Each pixel of the bimodal fusion matrices comprised a photodetector and a pressure sensor (referred to as VH unit), whereas the unimodal matrix included pixels with only a single type of sensor, either a photodetector or a pressure sensor. A convolutional‐like operation was executed by merging “n” pixels (where “n” denotes the kernel size) along each line in series to the iontronic synaptic transistor via the ionic cable. The unimodal matrix tended to lose essential features during multi‐transparency pattern mapping, while the bimodal fusion matrices could effectively extract both shape and transparency parameters of the patterns as depicted in Figure 6b. After the convolution operation, although the spatial integration resulted in some loss of pattern features, the recognition accuracy of the bimodal fusion surpassed that of the unimodal system (Figure 6c), which underscores the effectiveness of bimodal fusion in pattern recognition.

Besides multimodal sensing, crossmodal recognition enables humans to recognize and associate information between different sensory modalities such as generating a visual image from tactile input or imagining taste and smell from auditory descriptions. Multimodal artificial sensory system with crossmodal recognition facilitates the capacity of the network to interpret and link sensory information across different modalities. This enables modality‐independent identification^[^ ^117^ ^]^ and representation of multimodal information. Recently, a bioinspired multisensory neural network (MSeNN), integrated with not only multimodal sensors for crossmodal recognition but also spike encoding for efficient data processing, has been developed.^[^ ^49^ ^]^ As illustrated in Figure 6d, the MSeNN network utilized Si‐based photodetectors for vision, MXene‐based pressure sensors for touch, and sound detectors for hearing, respectively. The photomemristors in the system played an important role in integrating optical spikes from the various sensors and processing multisensory data. The architecture of the system facilitated supervised training of ANNs, providing associations between the five senses for high‐level cognitive capabilities. For instance, in the MSeNN‐based tactile‐visual system, letters were written by hand onto a 5 × 5 pressure sensor array and processed with five photomemristors (one per sensor row). The spiking outputs from these photomemristors during handwriting served as inputs to the ANN. The conducted training was integrated with tactile inputs with vision memory from optical projections of alphabet letters onto a 5 × 5 photodetector array (Figure 6e and second row of Figure 6f). After the training, the system can recognize and visually reproduce handwritten letters with 92% accuracy (fourth row of Figure 6f), demonstrating the effective integration of tactile and vision information processing. Additionally, the ANN can process auditory inputs and reproduce them into associated visual, olfactory, and gustatory outputs. Audio inputs were captured and transformed into 39‐dimensional vectors via Mel spectrograms, as well as vision, smell, and taste data were encoded into 12‐dimensional vectors via autoencoders. These vectors trained the ANN, enabling multisensory reproduction of images, smells, and tastes upon hearing a description (Figure 6g). Beyond recognition, the system also exhibited a form of artificial imagination where a never‐seen image of a blue apple was imagined based on the colors and the fruits experienced previously (Figure 6h). This design ensures efficient processing and interaction of diverse sensory data within the neural network.

Multimodal artificial sensory systems have usually employed separated sensors and signal processing units to detect and process various sensing signals into voltage spikes.^[^ ^49^, ^116^ ^]^ However, this design strategy leads to limited data conversion and movement, data latency, and high‐power consumption.^[^ ^68^ ^]^ Therefore, developing a multimodal artificial sensory system without the separation of sensing and processing components is highly desired. Recently, a MXene‐based vertical tribo‐transistor (VTT) integrated with a self‐powered TENG, possessing multi‐sensing, memory and computing capacities in the single device, has been constructed.^[^ ^118^ ^]^ As shown in Figure 6i,j, an open‐circuit voltage was generated via electrostatic induction when the gate electrode and the iongel layer (functioning as a triboelectric material) were brought into contact and then separated, demonstrating its self‐powering capacity. Multimodal sensory signal inputs (auditory and visual) were processed via the TENG component (serving as the sensor unit) and convolutional layers (acting as filters) to extract features for accurate emotion recognition. EPSC changes under various light intensities were categorized into 16 states, corresponding to colorful images for input layer processing. Data‐level fusion combined most original data, enabling recognition of basic emotions such as sadness fear, disgust, surprise, excitement, and anger through utilizing the multi‐source data analysis (Figure 6k). By fusing data from the multiple sensory modalities, the VTT can achieve a higher accuracy in emotion recognition compared to systems relying on a single sensory input (Figure 6l). The integration of sensing, memory, and computing into the monolithic device addresses key challenges in electronic device design and opens up new avenues for the development of intelligent, energy‐efficient technologies. The multimodal and crossmodal systems demonstrate an advanced level of sensory integration and cognitive processing, emulating human‐like capabilities in robotics and AI. This shows how different sensory inputs can be effectively combined and interpreted, paving the way for more intuitive and interactive robotic systems.

## 5 Sensory Robotics Based on Neuromorphic Perception

**Figure 7:**
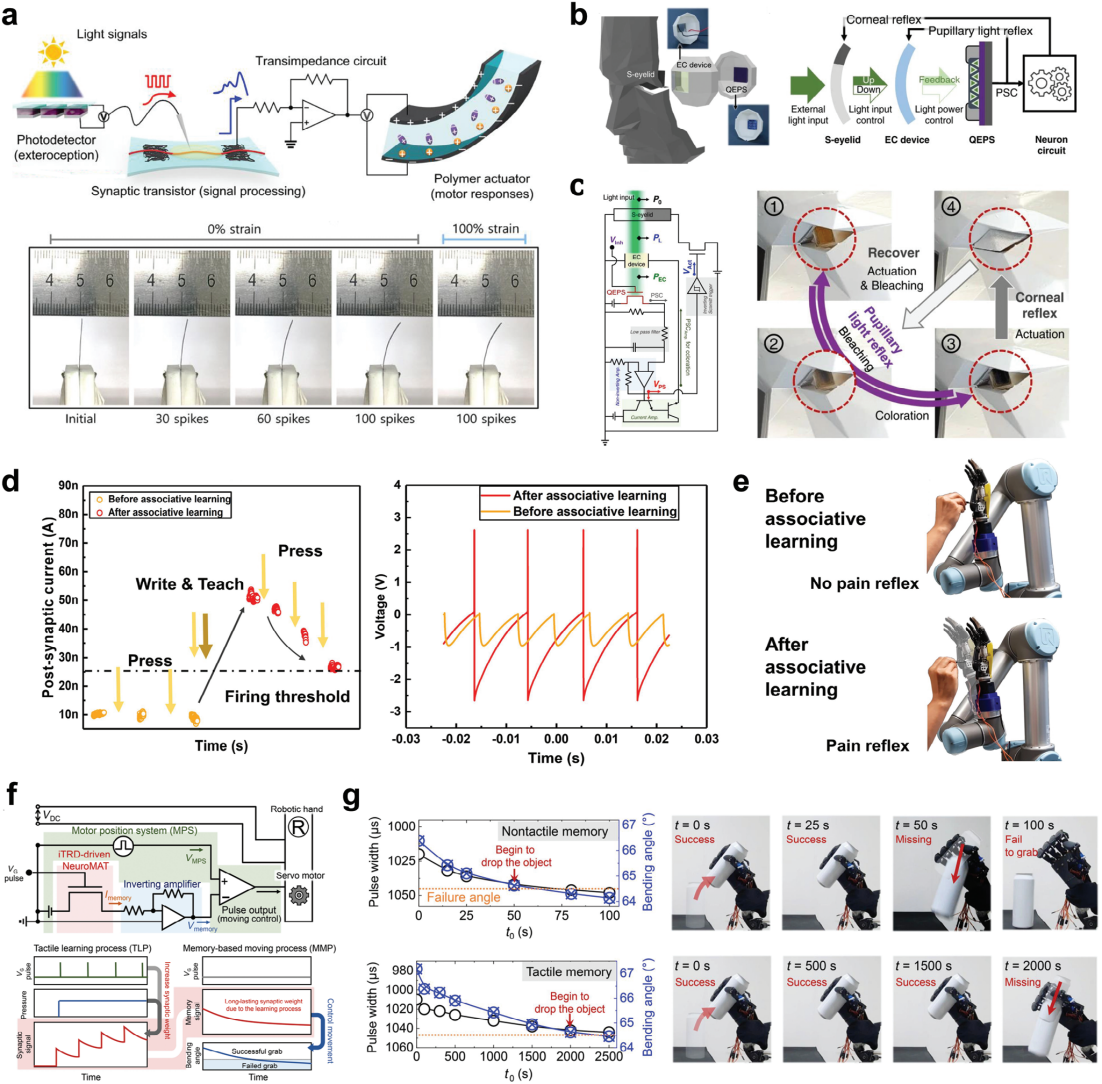
Application of NAS systems in sensory robotics a) Configuration of light‐sensitive sensorimotor nervetronics and photographs of the polymer actuator according to the presynaptic spikes with 0% or 100% strain. b) Schematics and signal flow of ocular prosthesis system. c) Circuit diagram and the photographs of pupillary light reflex and corneal reflex in operation. d) Illustration of the threshold of the computational e‐skin with a teacher signal and the firing pattern of the second‐order neuron before and after associative learning, under an applied force. e) Images depicting the acquired pain reflex after associative learning. f) Circuit diagram of the NeuroMAT‐based artificial tactile memory neuron. g) Photographs showing the variations in pulse width and bending angles during repeated gripping motion of a robotic hand in nontactile and tactile memory states, respectively. (a) Reproduced with permission.^[^ ^127^ ^]^ Copyright 2018, American Association for the Advancement of Science. (b,c) Reproduced with permission.^[^ ^128^ ^]^ Copyright 2022, Springer Nature. (d,e) Reproduced with permission.^[^ ^129^ ^]^ Copyright 2022, American Association for the Advancement of Science. (f,g) Reproduced with permission.^[^ ^68^ ^]^ Copyright 2023, American Association for the Advancement of Science.

Humans can sense, process neural signals, and effectively execute corresponding motor responses via the sensorimotor nervous system. The human sensorimotor nervous system offers a model for the design of neurologically inspired soft electronics,^[^ ^119^, ^120^ ^]^ artificial prosthetics,^[^ ^121^, ^122^, ^123^ ^]^ and neurorobotics.^[^ ^124^, ^125^, ^126^ ^]^ Consequently, the system can process neural information and produce synaptic response to perform human‐like motions. Emulating the human sensory and motor functions, an organic optoelectronic sensorimotor artificial synapse based on a stretchable organic nanowire synaptic transistor (s‐ONWST) has been reported.^[^ ^127^ ^]^ This neuromuscular system can detect and transmit optical sensory inputs, generating informative synaptic reactions and subsequent motor responses. The s‐ONWST was driven by voltage pulses from a self‐powered photodetector, producing synaptic outputs to mimic the actuation of biological muscles in artificial ones (**Figure** **7a**). Similarly, an ocular prosthesis system, integrated with quantum dot embedded photonic synapses (QEPS), an electrochromic (EC) device, and a solenoid‐based artificial eyelid (S‐eyelid), has been developed to mimic the autonomic pupillary light reflex and corneal reflex of human eyes (Figure 7b).^[^ ^128^ ^]^ Under incident light, the QEPS generated a PSC that causes the EC device to adjust its transmittance (transparency), changing the pupil size for the pupillary light reflex. Subsequently, the corneal reflex, represented by the S‐eyelid, responded to excessive light intensity by closing, akin to the natural blinking reflex of the eye, thus protecting the eye. This integration of the components enabled the ocular prosthesis to dynamically regulate incident light, protecting against high light intensities through mimicking the natural behavior of the human eye (Figure 7b,c). Moreover, to incorporate biological skin‐like haptic sensations into a robotic hand, a smart e‐skin that integrates printed synaptic transistor array using zinc oxide nanowires has been developed.^[^ ^129^ ^]^ Through an associate learning process, the presented device was equipped with a “teacher signal” that enables the e‐skin to adapt its response to stimuli based on experience. Without the teacher signal, the e‐skin was unable to learn and did not respond to the applied force. On the contrary, when the teacher signal was introduced, it facilitated localized “in‐skin” learning by enhancing the synaptic weights within the neural network of the e‐skin. This process facilitated a second‐order neuron (part of the tactile neural pathway) to transition from a non‐firing to a firing state in response to an over‐threshold stimulus (Figure 7d). As a result, the robotic hand acquired a pain reflex similar to pain reflex in human hands (Figure 7e), enhancing adaptability and intelligence for the prevention of excessive damage or injury. Likewise, to imitate memory‐based human motion, an advance system based on neuro‐inspired monolithic artificial tactile neuron (NeuroMAT)‐integrated neural circuit was designed by incorporating the NeuroMAT into an anthropomorphic robotic hand (Figure 7f).^[^ ^68^ ^]^ The NeuroMAT can enhance tactile perception and memory consolidation by utilizing iTRD mechanism in which the trap state of ions can be released by only tactile stimuli (as explained in Section 2.2). This approach enabled the robotic hand to acquire LTM of specific tactile motion task and replicate memory‐based human motion based on previous tactile experiences. As a result, the demonstrated system can perform reliable repeated gripping motion over time without failure, compared to the counterpart without tactile memory (Figure 7g). The successful implementation of artificial tactile nerve in robotic systems demonstrates their practical utility and potential for enhancing robotic dexterity and sensory perception, paving the way for future developments in neuro‐inspired robotics and artificial tactile sensing technologies.

## 6 Neuromorphic Neural Interface Systems

**Figure 8:**
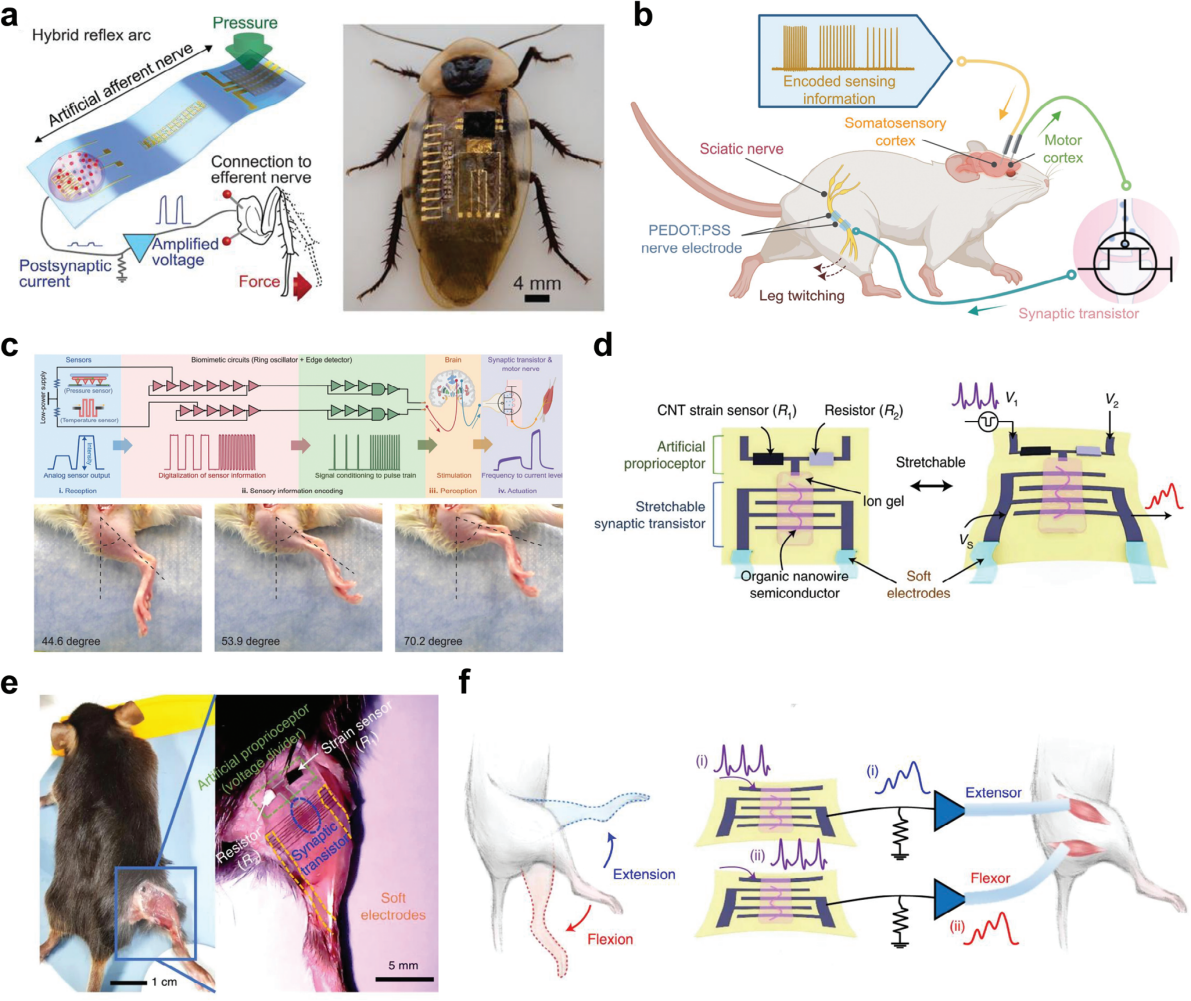
Application of NAS in neural interface systems a) Hybrid reflex arc made by integrating an artificial afferent nerve with a biological motor nerve (leg of a discoid cockroach). b) Schematic illustration showing the structure of the artificial sensorimotor system connected with a mouse sciatic nerve. c) Schematics of an artificial sensorimotor loop and photographs showing leg twitching angles in a mouse in response to different frequency stimulations correlating to different pressure inputs. d) Illustration of the structure and stretchable components of SNEN for proprioceptive feedback. e) Images of a mouse with SNEN attached to the leg to enable electrical stimulation of its extensor and flexors. f) Stimulation of the extensor and flexor of the leg with two artificial efferent nerves. (a) Reproduced with permission.^[^ ^41^ ^]^ Copyright 2018, American Association for the Advancement of Science. (b, c) Reproduced with permission.^[^ ^142^ ^]^ Copyright 2023, American Association for the Advancement of Science. (d‐f) Reproduced with permission.^[^ ^143^ ^]^ Copyright 2023, Springer Nature.

An artificial sensory synapse, which can detect and induce synaptic responses by controlling the frequency and intensity of stimuli,^[^ ^24^, ^130^, ^131^, ^132^, ^133^, ^134^, ^135^, ^136^, ^137^, ^138^ ^]^ facilitates the development of neural prosthetics that integrate artificial nerves with a living body in a hybrid system. This biohybrid system allows the artificial synapse to analyze sensory information through a mechanism like the one employed by biological sensory neurons to induce muscle movement.^[^ ^4^, ^139^, ^140^, ^141^ ^]^ In view of this, a hybrid monosynaptic artificial reflex arc that bridges an artificial afferent nerve with a biological efferent nerve from a discoid cockroach has been developed to emulate the biological reflex arc (**Figure** **8a**).^[^ ^41^ ^]^ The system detected pressure stimuli and processed the input information via the neuromorphic circuit, generating biomimetic postsynaptic signals. These signals were subsequently utilized to stimulate the biological efferent nerves in the leg of the cockroach, causing actuation of the tibial extensor muscle (Figure 8a). The capability of the system, modulating the movement and extension force of the leg in response to the intensity and duration of applied pressure, demonstrates its potential for advanced tactile sensory processing in biohybrid systems.

Moreover, to mimic both the sensory feedback and sensorimotor loop of the nervous system, an advanced monolithically integrated artificial synapse has been realized.^[^ ^142^ ^]^ The device integrated e‐skin with multimodal perception, neuromorphic pulse‐train signal generation, and sensorimotor closed‐loop actuation to generate nerve‐like pulse train signals in response to external stimuli. In the artificial sensorimotor loop, the e‐skin was connected to the somatosensory cortex of a rat which converts external stimuli into electrical pulse‐train signals. This caused evoked signals in the motor cortex to be transmitted via the artificial synapse to stimulate the sciatic nerve of the rat, causing downstream muscle actuation (Figure 8b,c). The system responded differently to varying degrees of mechanical forces, with the intensity of the motor response and subsequent muscle contractions correlating with the applied stimuli. Similar to the natural sensory feedback mechanism, the e‐skin system elicited larger leg twitching angles in response to stronger stimuli. On the other hand, a stretchable neuromorphic artificial efferent nerve (SNEN) that allows for proprioceptive feedback has been designed for neurorehabilitation purposes.^[^ ^143^ ^]^ The SNEN enabled the restoration of coordinated and smooth motions in limbs affected by neurological disorders by bypassing damaged neural pathways and directly stimulating muscles. The SNEN was composed of a stretchable synaptic transistor and an artificial proprioceptor (a strain sensor with a resistor) (Figure 8d). The stretchable synaptic transistors connected to both flexor and extensor muscles were stimulated alternatively to mimic biological natural movement. Additionally, the incorporation of the artificial proprioceptor allowed for the detection of leg movement while also preventing muscle overstretching by forming a closed feedback loop with the artificial synapse. This mechanism employed a negative feedback strategy, inspired by the biological muscle spindle, to regulate the EPSCs depending on the resistance changes of the strain sensor, ensuring muscle movements remained stable to prevent overstraining (Figure 8e,f). As the resistance in the strain sensor increased (i.e., overstretched muscle), the gate voltage decreased to adjust the EPSC generated by the transistor, modulating the muscle stimulation and effectively preventing overstretching. This feedback loop demonstrated the capability of the system to generate electrophysiological signals that mimic biological synaptic responses, enabling the precise control of muscle actuations in a live mouse. These controlled contractions facilitated natural movements like walking or running, demonstrating the potential of SNEN device in neurorehabilitation and the development of advanced prosthetic devices. It is noteworthy that the development of neuromorphic artificial interfaces highlights the significance of integrating sensory feedback into artificial nervous systems to restore complex motor functions, presenting a significant advancement in the field of bioengineering, robotics, and neuroprosthetics.

## 7 Conclusion and Perspective

The advancement of robotics and AI has significantly accelerated the design and development of NAS systems. These systems emulate the functionality and architecture of biological synapses and nervous systems. NAS systems with the ability to perceive and process sensory information like the neural pathways, not only detect various external stimuli but also convert these signals into transient channel currents via synaptic devices, thereby mimicking the somatosensory mechanisms found in biological sensory nerves.^[^ ^144^, ^145^, ^146^, ^147^, ^148^ ^]^ The integration of these synaptic signals with neural networks, governed by supervised or unsupervised machine learning algorithms, facilitates the direct processing of signals emanating from the diverse sensory inputs such as light,^[^ ^149^, ^150^, ^151^ ^]^ touch,^[^ ^152^, ^153^ ^]^ smell,^[^ ^28^, ^154^ ^]^ taste, and sound, enabling AI and robotics to perform complex recognition and decision‐making tasks. As highlighted in this review, significant advancements have been achieved in the development of NAS systems with various device structures and operational principles that effectively simulate several synaptic functions.

Specifically, the integration of artificial sensory nerves with biological efferent nerves also offers a novel approach for replacing damaged peripheral nerves and generating voluntary movements, providing a new standard for neuromorphic prosthetic devices. Consequently, artificial synapses can be engineered to produce synaptic responses indistinguishable from the electrophysiological signals of biological neurons. Incorporating a closed‐loop feedback mechanism, NAS systems could establish a real‐time adaptive feedback loop, autonomously adjusting neural interfacing activities to restore complex motor functions. These artificial systems with reliable synaptic outputs can serve as human‐machines interfaces, narrowing the gap between prosthetic devices and the biological nervous system. Ongoing research on nervetronics, encompassing neural‐inspired electronics such as neural prostheses, exoskeletons, and soft robotics that connect NAS systems with various living organs, are expected to accelerate due to rapid advancements in materials science, computer science, AI, healthcare, and synaptic devices.

Despite the progress discussed above, challenges in the practical application of NAS systems still exist, and further research investigations are needed. Here, we suggest several areas for future research.

1. Scalability: The fabrication of NAS systems often entails the use of separate sensors and processing units, which presents challenges in enhancing device density and achieving system‐level integration. In pursuit of energy‐efficient and compact NAS systems, strategies for monolithic integration, which minimize the distance between sensors and processing units, have been reported.^[^ ^66^, ^68^, ^108^ ^]^ Nevertheless, further research investigation on device‐device uniformity, stability, and other performance metrics are required to implement large‐scale integration.

2. Algorithm improvement: The processing of sensory information in NAS systems integrated with neural networks typically entails tasks such as recognition and classification, facilitated by the input of pre‐processed sensory information.^[^ ^155^, ^156^ ^]^ This functionality enables NAS systems to execute decisions or take actions based on the interpretation of sensory inputs. However, this intelligent capability is highly dependent on large amounts of sensory data, rendering the training process time‐consuming and susceptible to overfitting. To address these challenges, the technique of neural network pruning granularities can be leveraged to enhance computational efficiency and accelerate training time and help mitigate overfitting.^[^ ^157^ ^]^ This technique involves reducing the size and complexity of neural network models by eliminating less critical components, such as neurons, weights, or layers, without significantly degrading the network performance.^[^ ^157^ ^]^

3. Power consumption: Innovative strategies, such as iontronics, have been employed to realize low power consumption in synaptic devices. Nonetheless, the incorporation of machine learning algorithms and the separation of system components lead to substantial power consumption due to the extensive amount of energy required for data processing. Given that biological neurons demonstrate exceptionally low power consumption while maintaining high efficiency,^[^ ^4^, ^5^ ^]^ future development of NAS systems should integrate technologies that allow operation with minimal energy sources.

4. Memory retention: The biological neural system possesses the capability for both STM and LTM characteristics. Information in STM that is not actively rehearsed tends to diminish gradually, whereas repeated rehearsing transforms it into LTM. STM facilitates the removal of unnecessary information over time, whereas LTM enables the retrieval of information that has been stored for extended periods.^[^ ^6^, ^158^, ^159^ ^]^ The integration of STM and LTM functionalities is desirable in NAS systems, yet the memory retention abilities of existing NAS systems, particularly in terms of LTM, are inferior to their biological counterparts, with information loss occurring within a few hours. The development of advanced materials and design strategies is necessary to realize LTM retention capabilities that are on par with or exceed those of the biological neural system.

Research efforts to overcome these challenges will accelerate the advancement of next‐generation neuroprosthetics, nervetronics, and neurorobotics that are energy‐efficient and suitable for implantation. We believe that continued research into the development of high‐density iontronic neural device arrays^[^ ^160^ ^]^ could significantly diminish the physical dimensions of devices, enhance scalability, and reduce power consumption.

## Conflict of Interest

The authors declare no conflict of interest.
